# Exploring Owner Perceptions of the Impacts of Seasonal Weather Variations on Canine Activity and Potential Consequences for Human–Canine Relationships

**DOI:** 10.3390/ani11113302

**Published:** 2021-11-19

**Authors:** Emily J. Hall, Anne J. Carter, Mark J. Farnworth

**Affiliations:** 1 School of Animal, Rural and Environmental Sciences, Nottingham Trent University, Brackenhurst, Nottingham NG25 0QF, UK; anne.carter@ntu.ac.uk; 2 Department of Veterinary Health & Animal Sciences, Harper Adams University, Newport TF10 8NB, UK; MFarnworth@Harper-Adams.ac.uk

**Keywords:** climate change, human canine interaction, dog walking, canine sports

## Abstract

**Simple Summary:**

Dog walking is important for maintaining the good health and welfare of pet dogs. Environmental factors such as weather conditions have been previously identified as potential barriers to dog walking but, so far, focus has been on the impact this has on the human. With more seasonal weather variation predicted due to climate change, it is unclear if weather also impacts on the willingness of dogs to undertake exercise and thus the overall activity levels of pet dogs. An online survey recruited 3153 respondents to outline the impact of summer and winter weather conditions on daily canine activity levels. Owners reported their dogs were more impacted by cold (48.2% less likely to exercise their dog in the cold) and ice (64.0% less likely), than rain (25.3% less likely). In hot weather, 81.7% of owners reported reduced exercise duration and 87.0% reported less vigorous exercise by their dogs. As extreme weather events are likely to become more commonplace, it is likely this will negatively impact dog activity levels. Climate change mitigation strategies must therefore include considerations for dogs, if our canine companions are to retain their positions of service and companionship.

**Abstract:**

Climate change is leading to more instances of seasonal weather variation. Studies have explored the impact of adverse winter weather on dog walking, but the impact on the dog’s overall activity levels have not been previously considered. This study explored dog owner perceptions of the effects of both summer and winter weather on their dog’s activity levels. An international online survey recruited 3153 respondents between May and December 2018, to explore the impact of summer and winter weather conditions on baseline activity levels. Owners reported their dogs were more impacted by cold (48.2% less likely to exercise their dog in the cold) and ice (64.0% less likely), than rain (25.3% were less likely). In hot weather, over 80% of owners reported reduced exercise duration and vigour for their dogs. Carrying water or walking near water to facilitate activity in the summer was the most popular mitigation strategy (90.8%). Participation in dog sports appeared to reduce the impact of winter weather on canine activity and increase owner awareness of cooling strategies to facilitate summer activity. Strategies to promote safe activity participation are needed to maintain canine activity levels amidst rising global temperatures, including better understanding of cooling strategies for exercising dogs.

## 1. Introduction

Domestic dogs interlink with every aspect of human society, from companions to those working and providing assistance. Many of these roles depend on physical activity. Dog walking and canine sport participation are important components of the human–canine bond for pet dogs, whilst guiding, searching and even combat comprise key elements of assistance and service dogs’ duties. Extreme environmental conditions, particularly heat stress, affect both the performance ability and overall health of dogs, with heat-related illness reported to be the one of the most common causes of death for military working dogs [1]. Physical activity is a leading trigger of heat-related illness (HRI) in pet dogs living in the UK [2] and Israel [3]. Therefore, rising global temperatures have the potential to severely alter dogs’ roles in human society, posing a serious risk to both canine health and the human–canine bond, and thus canine welfare. Improving our understanding of this phenomenon has the potential to mitigate against harm, especially in an increasingly unpredictable environment [4].

Dog ownership likely has positive effects for owners. These include improvement in physical and mental health [5,6], including reduced depression, increased levels of oxytocin and decreased blood pressure and cholesterol levels [7]. Dogs also promote owners to exercise regularly, which decreases the risk of cardiovascular disease for both individuals [8,9]. Social contact amongst owners is facilitated by their dogs and decreases the feeling of loneliness [8]. Exercising with their owner is likely the primary source of exercise for a companion animal and so largely depends on owner-related factors such as their physical and social environment, own capabilities, preferences, motivations towards exercise and relationship with their dog [10]. In general, older adults are less likely to exercise in poor weather conditions, preferring warmer, dry days [11]. Ice, snow and rain have all been cited as reasons not to exercise in adults [12]. Dog owners also cited weather as a reason for reducing the frequency and duration of walks [13]. However, dog owners were more physically active than non-owners in poor weather conditions [14] when dog walking shifted from recreational to functional to fulfil the dog’s need for stimulation and exercise [15]. Whilst dog owners walk more times per week than non-owners, fewer than half of dog owners walk for a sufficient duration to obtain the recommended minimum of 150 min of moderate exercise a week [16,17]. Owners and dogs that share an active lifestyle complement each other, which results in high owner satisfaction [18]; owners that consider their dog to provide motivation and social support walk with them more frequently [19].

Dog walking also provides health benefits to dogs. Around half of the UK dog population is considered to be overweight or obese [20,21] and whilst diet is a major factor, exercise is also important. Overweight dogs receive, on average, fewer and shorter walks than dogs in ideal body condition [22,23]. Canine obesity is of increasing concern due to the risk of chronic diseases and reduction in the dog’s quality of life [24,25]. Increased exercise and dietary adjustments are the primary and most efficient methods of canine weight management [26]. Therefore, additional barriers to exercise such as weather conditions could potentially exacerbate the canine obesity epidemic.

Whilst studies have explored the impact of adverse, wet or wintery weather on owner’s frequency and duration of dog walks, there is a lack of information as to owners perceptions of their dogs’ ability to cope with seasonal weather variation. This includes both hot and cold extremes and how they impact on canine exercise levels. Additionally, previous studies mainly focus on dog walking as the form of canine activity; there are limited data available regarding the wider types of exercise undertaken. Hall et al. [2] identified that exercise is responsible for almost three quarters of all HRI cases in UK dogs, and over a third of brachycephalic dog owners report concerns about overheating [27]. Both brachycephalic dogs and obese dogs are at greater risk of developing HRI [28,29], raising concerns that the changing demographic of the pet dog population (rising levels of obesity and increasing popularity of brachycephalic breeds) could further negatively impact canine activity levels as climate change increases the frequency of seasonal weather variations. If dogs are to retain their many roles in human society, a better understanding of the impact weather extremes have on canine activity is needed.

This study aimed to explore dog-owner perceptions of the effects of both summer and winter weather on their dog’s activity levels. Using analysis of an international survey of dog-owners, we aimed to explore how canine factors such as breed, age, sex and skull shape influenced owner perceptions and the use of mitigation strategies to continue activity in adverse weather (e.g., dog coats and cooling strategies). An additional aim of this study was to report the variety of activities, and duration of activity regularly undertaken by owned dogs.

## 2. Materials and Methods

This study was approved by Nottingham Trent University School of Animal, Rural and Environmental Sciences Ethical Review Group under application ARE802.

### 2.1. Survey Creation and Distribution

An online survey for dog owners was created using JISC Online Surveys (Jisc, Bristol, UK). The survey was initially distributed in English, then additionally in Portuguese, Spanish, Dutch and Hungarian following offers of translations. The survey was distributed using social media (predominantly Facebook and Twitter) and was subsequently shared by the popular UK veterinary press (Vet Times and VN Times) between 25 May and 31 December 2018. The survey was open to all dog owners aged 18 years or older, and owners were asked to complete the survey for each of their dogs. The survey included the following sections: dog demographics, dog routine activity levels and sport participation, dog activity levels in winter, dog activity levels in summer and problems occurring during or following canine activity. Participants were not asked for any personal data in the survey, only their opinions and details relating to their dogs. Questions consisted of multiple- and single-choice questions, Likert scales, and open-ended free text questions. Only the sections relating to seasonal impact on activity are considered in this paper.

### 2.2. Questionnaire Design

The English version of the questionnaire was created iteratively, during which a small number of dog owners were recruited for pilot testing to ensure ease of use and clarity. The survey was presented in five sections, four of which are considered in this study.

#### 2.2.1. Demographic Data

Owners were asked their dog’s country of residence, age, sex and neuter status, breed type (purebred, designer hybrid or crossbreed) and bodyweight (kg). All breed types with at least 10 survey responses were taken forward for analysis as individual subgroups. A designer hybrid was defined as a named cross between known breeds with a contrived name, e.g., Cockapoo [28]. Breed types with fewer than 10 survey responses were combined into two groups: other purebred and other designer hybrid. From the owner-reported breed, dogs were classified by skull length (dolichocephalic, meaning long front to back compared to width, mesocephalic, meaning neither brachy- nor dolichocephalic, or brachycephalic, meaning short front to back compared to width) (see Hall et al. [28] for categorisation of skull shape). Categorical variables for age (eight sub-groups) and bodyweight (seven sub-groups including “missing” where no bodyweight was reported) were created for analysis. The distribution of sub-groups for age and bodyweight were selected to enable comparison to previous studies using veterinary clinical data [2,28]. The location (country) data were reported at the continent level to account for the relatively small number of responses from some countries.

#### 2.2.2. Normal Exercise Routine

Owners were asked to estimate their dog’s routine activity levels by stating the total time spent exercising outside the house per day and the frequency of active periods each day. The owner-reported activity levels of adult dogs (≥1 year) were compared to the UK Kennel Club [30] recommended daily activity levels. Owners also estimated annual frequency of participation in the following activities: off-lead exercise, high-intensity exercise, walking, running, swimming (indoors and outdoors), playing fetch/retrieving and canine sports.

#### 2.2.3. Winter Activity Levels

Using a Likert scale, owners reported the impact of cold, rain, and ice on their dog’s exercise duration (dog exercises much more; a little more; no change; a little less; much less). Owners were then asked a series of questions regarding the impact of winter weather on their own willingness to exercise the dog, or their perception of the dog’s willingness to exercise (Table 1). These questions were used to create a winter canine activity score (winter CAS), where the higher the score (maximum score 45), the greater the negative impact of winter on the dog’s activity. Owners were finally asked if they used a dog coat to facilitate exercise in winter using the same scale as the questions in Table 1.

#### 2.2.4. Summer Activity Levels

Using a Likert scale, owners reported the impact of heat and humidity on their dog’s duration and intensity of exercise (dog exercises much more; a little more; no change; a little less; much less). Owners were then asked a series of questions regarding the impact of summer weather on their willingness to exercise the dog, or their perception of the dog’s willingness to exercise (Table 2). These questions were used to create a summer CAS, where the higher the score (maximum score 45), the greater the negative impact of summer on the dog’s activity. Owners were finally asked three questions about the mitigations they use to facilitate exercise in summer (I carry water or walk near water to facilitate exercise, I use a cooling aid such as a cooling coat to facilitate exercise, and I cool my dog after exercise in the heat) using the same scale as the questions in Table 2.

A fifth survey section collected owner reports of problems their dog had experienced during or after exercise; this section is not considered in this study.

### 2.3. Data Analysis

Survey responses were exported to Microsoft Excel (v16, Redmond, WA, USA) for initial cleaning, removal of incomplete responses, calculation of descriptive statistics and to generate the summer and winter CAS. Statistical analyses were then performed using IBM SPSS Statistics v28 (IMB Inc., Armonk, NY, USA). The questions from Table 1 and Table 2 were explored using factor analysis to confirm they measured two separate constructs, summer and winter. The internal consistency of the questions comprising the summer and winter CAS was then determined using Cronbach’s alpha, with >0.7 considered acceptable. The summer and winter CAS were analysed as continuous variables and were found to have non-parametric distributions following visual assessment of histograms and the Shapiro–Wilk test. Linear regression was used to determine which variables (skull shape, age, bodyweight, sex/neuter, sport participation, daily exercise, continent) were associated with increased summer and winter CAS. Due to the relatively low numbers of some breed types, this variable was included for univariable exploration only for winter and summer CAS and coat use in winter, and results are presented in the Appendix A and Appendix B. Binary logistic regression was used to determine which variables were associated with the use of winter and summer activity mitigation actions to facilitate exercise, the scaled responses were converted to binary outcomes of agree or did not agree. Variables with liberal associations in univariable tests (*p* < 0.2) were taken forward for multivariable evaluation using generalised linear models, a gamma distribution was used to analyse the summer and winter weather effect on summer and winter CAS, and binary logistic modelling was used to analyse the use of mitigation actions. Model development used backwards stepwise elimination and Akaike information criterion (AIC) for model selection (with lower values indicating better model fit) with graphical analysis of the residuals to assess for goodness of fit. The use of stepwise model development is not ideal; however, the aim of this analysis was to generate exploratory rather than predictive models. The area under the receiver operating characteristic (ROC) curve and Hosmer–Lemeshow (HL) test were used to evaluate the explanatory ability of the binary logistic models [31] alongside consideration of the underpinning biological plausibility of the model specification. Statistical Results are reported as mean ± standard deviation (SD) for variables with a parametric distribution, and median with interquartile range (IQR) for data with a non-parametric distribution. A *p*-value of <0.05 was considered significant.

## 3. Results

### 3.1. Canine Demographic

In total, 3153 responses were received from owners of dogs from 222 breed types, including 189 purebred breeds, 33 designer hybrid types and crossbreeds. The majority of dogs had a mesocephalic skull shape (*n* = 1962, 62.2%), followed by dolichocephalic (*n* = 386, 12.2%), brachycephalic (*n* = 188, 6.0%) and brachycephalic cross (*n* = 59, 1.9%); 558 (17.7%) of the dogs’ skull shapes were not known (e.g., crossbred dogs). Dogs were predominantly from Europe (*n* = 2660, 84.4%), followed by North America (*n* = 319, 10.1%), Australia (*n* = 114, 3.6%) and Asia (*n* = 35, 1.1%); less than 1% were from South America and Africa.

The median age of dogs in the study was 5.0 years (IQR 2–8 years, overall range: 3 months to 20 years). In the study population, 1662 (52.7%) dogs were male, and 920 (29.2%) dogs were entire (518 (16.4%) and 402 (12.8%) male and female, respectively). The median reported bodyweight was 21 kg (IQR: 13–28 kg, overall range: 1–89 kg).

### 3.2. Routine Activity Levels

**Figure 1:**
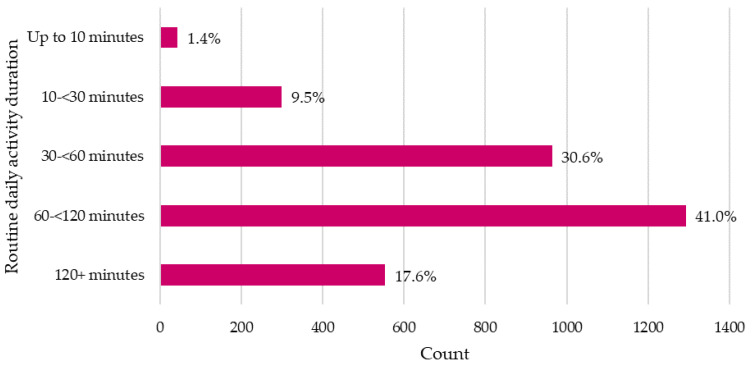
Distribution of daily activity duration for dogs reported by an owner survey.

The majority of dogs completed over 60 min of activity per day (58.6%), whilst 10.9% of dogs completed under 30 min of activity per day (Figure 1). Most dogs were active multiple times per day: two activity sessions per day (45.5%), three activity sessions per day (20.6%) and four or more activity sessions per day (5.4%), whilst 27.1% had only one activity session per day and 4.3% had “other” activity routine (i.e., “house dogs” undertaking no outdoor activity; dogs living outdoors with free access to an enclosed space for activity; dogs spending all day walking or working with their owner).

The comparison between the reported routine daily activity levels of dogs from the survey with the UK Kennel Club breed activity recommendations are shown in Table 3. Of 1713 dogs aged 1 year or over from the listed breeds, 60.8% did not achieve the recommended daily activity duration and 14.8% exceeded the recommendation. For breeds with a daily recommendation of more than 120 min of activity per day, 77.4% of adult dogs included in the survey did not meet this recommendation. The breeds with the highest percentage of responses meeting the recommendation were the Dalmatian, German Shepherd Dog and Weimaraner. Only 5.8% of Golden Retrievers met the recommendation and none of the Rottweilers in the survey met the recommendation. For dogs with a daily recommendation of 60 min of activity per day, over 50% of the Beagles, Border Terriers, Cocker Spaniels, Dachshunds, Greyhounds and Shetland Sheepdogs in the survey exceeded this recommendation, whilst 33.3% of Cavalier King Charles Spaniels and 28.6% of Pugs did not achieve this recommendation. Of the three breeds with a recommendation for 30 min of activity per day, 73.9% of Papillons and 46.7% of Yorkshire Terriers exceeded this, whilst 21.4% of Chihuahuas did not meet this recommendation.

The frequency and types of activity undertaken by the dogs included in the survey are shown in Table 4. Over half (52.1%) the dogs in the survey completed high-intensity exercise on a daily basis, 38.1% swam outdoors at least once per month whilst 4.8% swam indoors or attended hydrotherapy at least once per month, and 24.9% participated in a canine sport at least once per month. For 42.4% of dogs playing fetch/retrieving was a daily activity, 75.3% of dogs in the survey played fetch/retrieved at least monthly.

Of the 873 dogs participating in canine sports, 844 responses listed the specific sports. The majority of those dogs participated in one sport, although 193 (22.9%) were reported to participate in more than one sport, with one dog participating in six different sports. The most commonly listed sport was agility (44.4%), followed by sled sports (including both dryland disciplines such as canicross and traditional mushing disciplines) (25.0%), gundog work including field trials (18.4%), scent work and tracking (12.8%) and obedience and protection disciplines (11.1%).

### 3.3. Owner Perception of Winter Weather Effects on Canine Activity Levels

Overall, owners reported cold and ice had more impact than rain, with 48.2% less likely to exercise their dog in the cold, 64.0% less likely to exercise in icy conditions and 25.3% less likely to exercise in the rain. Conversely, 19.8% were more likely to exercise in rain, and 2.6% and 3.1% more likely to exercise in cold and ice, respectively.

Multivariable generalised linear modelling identified seven variables that were significantly associated with the winter CAS including skull shape, bodyweight, age, sex/neuter, sport participation, daily exercise and continent (Table 5). Mesocephalic dogs had lower winter CAS than all other skull shapes, indicating that mesocephalic dogs’ activity levels were less impacted by winter. Dogs weighing under 10 kg had significantly higher winter weather effects scores compared to dogs weighing 10 kg or over. Dogs aged 10 years or over had significantly higher winter CAS compared to dogs aged less than 2 years. Male and female neutered dogs tended to have higher winter CAS compared to entire female dogs. Dogs that participated in sport were considered to be significantly less affected by winter weather compared to dogs not participating in sports. Dogs completing 60 min or more exercise each day had lower winter CAS compared to dogs completing less than 10 min of exercise each day. Dogs living in Australia, North America and South America were considered significantly less affected by winter weather compared to dogs living in Europe. Univariable results for breed type are presented in Appendix A. Compared to Labrador Retrievers, 18 breed types were considered significantly more affected by winter weather (notably the Yorkshire Terrier, Whippet, Chihuahua, Greyhound and Pug), and three breed types were considered significantly less affected by winter weather (Siberian Husky, Working Cocker Spaniel and Springer Spaniel) (Table A1).

### 3.4. Owner Perception of Summer Weather Effects on Canine Activity Levels

Overall, 81.7% of owners reported that hot weather reduced their dog’s exercise duration and 87.0% reported that hot weather reduced how vigorously their dog could exercise. Similarly, 79.6% and 81.5% of owners reported that high humidity reduced exercise duration and vigour, respectively. Conversely only 3.9% and 2.5% of owners reported that hot weather increased their dog’s exercise duration and vigour, and only 1.9% and 2.0% of owners reported that high humidity increased their dog’s exercise duration and vigour (respectively).

Multivariable generalised linear modelling identified six variables that were significantly associated with perceived summer weather effects on CAS including skull shape, bodyweight, sport participation, sex/neuter, daily exercise and continent (Table 6). Dogs exercised one hour a day or more were considered to have significantly lower summer CAS than dogs exercised under 10 min per day, and dogs living in Asia were considered to have significantly higher summer CAS than dogs living in Europe, who in turn had significantly higher summer CAS than dogs living in Australia. Brachycephalic dogs were considered to have significantly higher summer CAS than mesocephalic dogs, and dogs weighing 30 kg or over were considered to have significantly higher summer CAS when compared to dogs weighing under 10 kg. Dogs that participated in sports were considered to have significantly lower summer CAS, and compared to entire female dogs, neutered male dogs were considered to have higher summer CAS (Table 6). Univariable results for breed type are presented in Appendix A. Compared to Labrador Retriever, three breed types were considered to have significantly lower summer CAS (Springer Spaniel, Collie and Pointer (English)) and three breed types were considered to have significantly higher summer CAS (Boxer, French Bulldog and Staffordshire Bull Terrier) (Table A2).

### 3.5. Use of Mitigation Strategies to Facilitate Exercise in the Winter

Overall, 50.0% (1565/3128) of owners agreed that they use a coat in order to continue winter activity (modal response strongly disagree). The variables breed type (*p* < 0.001), sex/neuter (*p* < 0.001), age (*p* = 0.130), skull shape (*p* < 0.001) and bodyweight (*p* < 0.001) were associated with the use of a coat in winter. The variables sport participation, daily exercise and continent were not associated with coat use in winter (*p* > 0.2). The final model retained two variables, skull shape and bodyweight (Table 7) and showed acceptable discrimination (HL = 0.851, area under the ROC curve: 0.627). Compared to dogs with a mesocephalic skull shape, all other dogs had significantly greater odds for coat use in winter; brachycephalic designer crossbreeds had the greatest odds (OR 3.66). Compared to dogs weighing under 10 kg, all other dog sizes had significantly reduced odds for coat use in winter; dogs weighing over 50 kg had the lowest odds (OR 0.21). Univariable logistic regression results for breed type are included in Appendix B. Compared with the Labrador Retriever, 22 breed types had significantly greater odds of coat use in winter; the highest coat use was reported for Whippets (OR 26.69), Greyhounds (OR 14.18) and Cavalier King Charles Spaniels (OR 13.35). Golden Retrievers (OR 0.48) had significantly lower odds for coat use (Table A3).

### 3.6. Use of Mitigation Strategies to Facilitate Exercise in the Summer

Carrying water or walking near water to facilitate activity in the summer was the most popular mitigation strategy, with 90.8% (2854/3143) of owners agreeing to its use (modal response: strongly agree). As the majority of respondents reported using this mitigation strategy, further analysis was not performed.

Overall, 58.6% (1834/3130) of owners agreed that they cool their dog after exercise in the heat (modal response: strongly agree). All variables were liberally associated with cooling after exercise (*p* < 0.2), and the final model retained four variables: skull shape, age, daily exercise and sports participation (HL = 0.777, area under the ROC curve: 0.601). Dogs aged 2 to <14 years had significantly lower odds for cooling after exercise compared to dogs aged under 2 years. Dogs with no skull shape available (crossbreeds) had significantly reduced odds for cooling compared to mesocephalic dogs. Dogs completing under 60 min of exercise per day had significantly reduced odds for cooling compared to dogs completing 120 min or more, and dogs participating in sports had 1.45 times the odds of cooling compared to dogs not participating in sports (Table 8). Univariable logistic regression results for breed type are included in Appendix B. Compared to the Labrador Retriever, two breed types had significantly greater odds of cooling post exercise, French Bulldogs (OR 7.92) and Springer Spaniels (OR 1.75). Crossbreeds, other purebreds, Jack Russel Terriers, Yorkshire Terriers, Cavalier King Charles Spaniels and Shih-Tzus had significantly lower odds for post-exercise cooling (Table A4).

The least commonly employed mitigation strategy was the use of cooling aids (such as cooling coats); only 25.4% (792/3121) of owners agreed that they used a cooling aid to facilitate activity in summer (modal response strongly disagree). All variables were associated with use of a cooling aid in summer (*p* < 0.2), and the final model retained two variables: sports participation and skull shape (HL = 0.929, area under the ROC curve: 0.652). Dogs participating in canine sports had 2.05 times the odds for cooling aid use to facilitate exercise in the summer compared to dogs not participating in sports, and brachycephalic dogs had 1.98 times the odds for cooling aid use compared to mesocephalic dogs (Table 9).

## 4. Discussion

This study reports owner perceptions of their dog’s routine activity levels and how seasonal weather extremes (summer and winter) and canine factors impact upon them. Dog owners’ use of mitigation actions such as canine coats, cooling aids and post-exercise cooling are also reported. Dependent on current practices, these could form the basis of educational campaigns to support adaptive responses to seasonal weather variations in order to maintain canine activity. It is important to distinguish that whilst previous studies have looked at the impact of weather conditions as a factor affecting owner desire to walk and subsequent effects on owner activity levels, this study focused on the perceived impact of weather on the dog’s activity levels, including both activity associated with the owner and activity independent of the owner.

Whilst the level of exercise required by a dog varies according to a number of factors (e.g., breed; age; health status; size), it is recommended that even the smaller breeds (e.g., Chihuahuas and Papillons) receive at least 30 min of exercise a day, split over a number of sessions [23,30]. Pickup et al. [23] reported that whilst there are no evidence-based guidelines on individual breed exercise requirements, only 50% of dogs surveyed received the Kennel Club recommended daily activity for that breed. In the present study, only 39.2% of adult dogs from the breeds reviewed met the recommended daily activity, with fewer than 6% of Golden Retrievers and no Rottweilers achieving the recommendation. Larger and energetic breeds are more likely to be walked for longer [32]. However, the findings of this study show that 10.8% of the dogs surveyed received less than 30 min of exercise per day and 27.1% were only active for a single period.

Typically, the activity levels of family dogs are largely controlled by owners and result in just one period of higher-intensity daily activity. Conversely, farm dogs and free-roaming dogs display two distinct periods of activity with longer duration, but lower intensity. [10]. This finding is reflected in the present study, over half of the dogs surveyed completed high intensity exercise on a daily basis. The most frequently selected daily activity for the dogs in the current study was walking off lead (64.4%) followed by running off lead (59.9%) then walking on lead (47.7%). Fetch and retrieving activities featured as a daily (42.4%) or frequent (at least monthly) activity for 75.3% of dogs. Retrieving activities, particularly ball throwing, can be used as a means of exercising dogs with limited requirement for the owner to move [8]; however, concerns have been raised regarding the impact of repetitive ball throwing both behaviourally and due to the potential for repetitive strain injuries [33] and exacerbation of osteoarthritis [34].

As the majority of pet dogs are not receiving the recommended levels of daily activity, and around half of the UK pet dog population is reported to be overweight [20,21], it is concerning that over 80% of survey respondents in the present study reported that hot summer weather reduced both the duration and vigour of their dog’s activity. Due to the subject of the survey, it is probable that the participants are more engaged with their dog’s activity levels, meaning these results likely reflect a population of dogs that are more active than the general population. Less active dogs will suffer greater impacts of extreme heat, as reduced activity impairs thermoregulation during exercise [35]. As global temperatures increase further, this finding suggests that the majority of pet dogs will experience reduced activity levels due to hot weather. Obese dogs are at greater risk of HRI [28,29], including specifically increased risk for exertional HRI [2], creating a potentially lethal spiral of rising temperatures resulting in more obese dogs that are at greater risk of exertional HRI when they do manage to exercise. It is perhaps unsurprising that many dog owners are already reporting using mitigation actions to continue exercising their dogs in hot weather.

The summer CAS variable attempted to measure owner perceptions of the impact extreme summer weather (heat and humidity) has on canine activity, with higher summer CAS scores inferring greater negative impacts on the dog’s ability/willingness to exercise in hot weather. The variables skull shape, bodyweight, sports participation, sex/neuter, daily exercise, and continent were found to be associated with summer CAS. At univariable levels, three breeds (Pointer (English), Collie and Springer Spaniel) were found to have lower scores, and three breeds (Boxer, French Bulldog and Staffordshire Bull Terrier) were found to have higher scores compared to Labrador Retrievers. This result is concerning, as Springer Spaniels and Collies were previously found to be at greater risk of exertional HRI than Labrador Retrievers in UK dogs presenting to veterinary clinics with HRI [2]. This suggests there may be a mismatch between the owner’s perception of their dog’s ability to exercise in hot weather and the dog’s actual thermoregulatory ability. Conversely, male neutered dogs were considered to have higher summer CAS compared to female entire dogs, which reflects the findings of Hall et al. [2], that male neutered dogs had the greatest odds for exertional HRI, and studies report higher post-exercise body temperatures in male dogs compared to females [36,37]. Dogs active for 120 min or more each day were considered to have lower summer CAS compared to dogs completing less than 10 min per day. This could be because dogs completing over 120 min of daily activity are exercising less intensely and are therefore less affected by heat. Alternatively, the most active dogs are simply physically fitter, and able to better tolerate exercise in hot environments. As improved fitness has been found to improve thermoregulation during exercise [35], the latter is potentially more likely.

Dogs living in Australia were considered to have lower summer CAS compared to dogs living in Europe. Whilst the number of survey respondents from Australia compared to Europe must be considered here, this result is somewhat unexpected given the prolonged summer heat experienced by much of the continent and increasingly frequent extreme heat events. This finding could indicate a mismatch between Australian owners’ perceptions of their dog’s ability to exercise in the heat, and the dog’s actual tolerance of summer conditions. Reports of animals left in hot cars in Australia increased year on year from 2008 to 2018 [38], with suggestions that owners of adult dogs may imagine that their dog is more able to tolerate heat than they actually are. Given the increasing frequency of extreme heat events in this region, these findings suggest further research into owner awareness of heat-related illness is urgently needed.

The results of the present survey suggest that hot weather is already negatively impacting canine activity levels, so mitigation strategies may be important to increase activity and reduce obesity. This is especially important given that temperatures are likely to increase in the near to long term. Over 90% of dog owners in the present study reported carrying water or exercising in close proximity to water to facilitate dog activity in the summer. Additionally, 23.7% of respondents reported that their dog swims outdoors at least once a week as a baseline. Two other mitigation actions for exercising dogs in hot weather were explored in the present study. Over half of the respondents agreed that they regularly cool their dog after activity in hot weather, with skull shape, dog age, daily activity levels and canine sport participation identified as factors associated with this practice. Notably, owners of French Bulldogs had almost eight times the odds for post-exercise cooling compared to owners of Labrador Retrievers, and owners of dogs participating in canine sports had 1.45 times the odds for post-exercise cooling use compared to non-sporting dog owners. French Bulldogs have been found to have increased risk of exertional HRI [2], so this increased use of cooling aids could simply reflect owners’ awareness of their dog’s thermoregulatory problems. However, social influences such as fashion and media attention are thought to have had a role in the soaring popularity of this breed [39,40,41], with their distinctive appearance making them popular with users of social media despite their many health problems [42]. The increased use of cooling aids amongst both French Bulldog owners and sports dog owners could similarly reflect the influence of social media, with owners sharing management tips and photos of their dogs using such devices to breed/sport groups and sharing anecdotal evidence of their effectiveness. Owners of dogs aged under 2 years old and dogs active for 120 min or more per day had the highest odds for post-exercise cooling, likely reflecting the high-energy behaviour of young, fit and active dogs and their increased risk of exertional HRI compared to older animals [2]. This finding is positive, suggesting that awareness of the risk of HRI following exercise is improving amongst dog owners, but as exercise is the leading trigger of HRI in dogs [2], continued owner education is essential. Historically, concerns were raised regarding the use of cold water for cooling dogs affected by HRI and myths persist that promote the use of ineffective cooling methods such as putting alcohol on paws [43]. However, both human and canine studies have shown that evaporative cooling using (preferably cold) water and air movement or cold water immersion are the most effective methods of cooling dogs in an emergency situation [44,45,46,47,48].

Only a quarter of the survey respondents reported using a cooling aid such as a cooling coat to facilitate dog activity in hot weather. Owners of dogs participating in canine sports, and brachycephalic dogs both had around two times the odds for cooling aid use compared to non-sporting dogs and mesocephalic dogs, respectively. These results support the findings reported by Packer et al. [27], that over a third of brachycephalic dog owners recognized that their dog had a problem with heat regulation. Not only are owners of brachycephalic dogs more likely to recognize thermoregulation problems (brachycephalic dogs were also considered to have higher summer CAS), they also appear to be more likely to utilize cooling aids such as cooling coats to facilitate activity in summer. This is concerning, as there is a paucity of peer-reviewed evidence relating to the effectiveness of canine cooling coats, and similar cooling aids (moistened multilayer breathable fabric coats) have been found to be ineffective when used on exercising horses [49]. Continued use of ineffective cooling aids could inadvertently put more dogs at risk of HRI, if owners believe the use of such devices could mitigate hot weather that would otherwise prevent them from exercising their dog.

Owners of dogs competing in canine sports reported significantly lower winter CAS, suggesting their dogs were less likely to experience activity disruption due to wet and cold weather. Whilst they also reported significantly reduced summer CAS, they were also more likely to cool their dog after exercise in hot weather and use cooling aids (such as cooling coats) to facilitate exercise in hot weather. Previous studies have reported dog owner motivations for competing in canine sports, including connection to the dog, physical activity for the dog and owner, and the learning and enjoyment that comes from training [50]. Owners of dogs participating in sports may be less likely to neuter their dog [51], which could explain why neutered dogs tended to have higher winter CAS scores than entire dogs. Perhaps unsurprisingly, obesity levels in dogs competing in canine sports were lower than pet dogs not participating in sports in a study by Kluess et al. [51]. This was attributed to better owner awareness of body condition scoring, lower overall rations fed and fewer treats fed, and higher levels of engagement with activities that required owner involvement. The results from the present study add further evidence to sports-dog owners having increased motivation to exercise, even in poor weather, and suggest that sports-dog owners may be more aware of the potential risk associated with canine activity—notably HRI—and have better awareness of the mitigation actions that can be employed to maintain activity in hot weather. As early cooling has been found to improve survival in dogs affected by HRI [52], improving owners’ ability to recognize early signs of HRI and dispelling myths associated with effective cooling methods (notably outdated concerns relating to the use of cold water) should be considered educational priorities for dog owners in the face of rising global temperatures [53].

As previously reported, inclement winter weather conditions can reduce owner motivations to exercise their dog [54]. The present study found that owners considered their dog’s activity to be reduced by icy conditions (64.0%) more than cold (48.2%) or rain (25.3%). These findings likely reflect the owner’s willingness to brave winter weather conditions more than the dog’s willingness, as previous studies have reported that neither rain nor snow had a noticeable impact on free-roaming urban dogs, with dog activity increasing in response to cloud cover [55]. Whilst rain increased exercise levels for almost a fifth of dogs (19.8%), cold and ice had little positive impact on increasing exercise in any of the dogs (2.6% and 3.1%, respectively). Whilst icy conditions are less common than other winter weather, they are arguably harder to mitigate in terms of likelihood to slip, fall or receive a pad injury.

Half of owners reported using a coat on their dog in winter to continue exercising, with owners of breeds with less body fat (Greyhound and Whippet), thinner coats (Pointer and Boxer) and small breeds (Chihuahua, Cavalier King Charles Spaniel and Pug) most likely to use them. The dogs considered to have the lowest winter CAS (and therefore more likely to exercise more in cold, wet and icy weather) were those with a normal exercise period of 60 min or more. This means that dogs with the shortest regular activity duration are more likely to miss out on opportunities to exercise in the winter, when it is potentially safest for them to do so. In addition, older dogs (>8 years) were considered to be more negatively affected by adverse weather than those over 2. Small dogs (under 10 kg) were considered to be more negatively affected by adverse weather, despite having the greatest odds for coat use in winter. Coat use may also be related to fashion and the anthropomorphism of small dogs [56,57], rather than being purely functional to facilitate exercise.

Dogs with a brachycephalic skull shape and designer hybrids with a brachycephalic skull shape within the breeding had the greatest odds for coat use in winter. These dogs were also perceived to have higher winter CAS, meaning they likely experienced reduced activity opportunities in winter despite their coat use. Some brachycephalic breeds including Pugs and Cavalier King Charles Spaniels are reported to be at increased risk of being overweight [58], meaning it is unlikely they need an additional coat in winter, and would in fact probably benefit from the additional activity, provided their respiratory function allows them to exercise safely [59].

The perceptions of dog owners reported in the present study suggest increasing seasonal weather variation will have profound effects on the activity element of human–canine relationships in the future. With over 80% of owners reporting that hot and humid weather reduces their dog’s summer activity, global warming could seriously impact the way owners interact with their dogs, with lower levels of activity potentially worsening the canine obesity epidemic. Worryingly, there appears to be a disparity between some dog owners’ perceptions of their pet’s ability to exercise in hot weather, and the dog’s potential risk of HRI, and a quarter of the owners surveyed reported using cooling aids such as cooling coats to facilitate exercise despite there being no good evidence to support their effectiveness. As exercise is an important trigger of HRI [2,3], it is vital that dog owners receive evidence-informed advice to support safe exercise practices in hot, summer weather. Free-roaming urban dogs and free-roaming owned dogs have been found to limit their activity to early mornings and later evenings to avoid the heat of the day in hot weather [10,55], and similar patterns of dog walking and exercising need to be adopted by owners. However, having to rise early to exercise a pet dog may create a barrier to pet ownership, and pose particular challenges to women who walk alone due to the potential risk of being attacked if walking alone in lowlight conditions. Failure to adapt to rising global temperatures and allowing canine activity levels to drop risks a worsening of the canine obesity crisis with associated health impacts [26], and potentially increased levels of behavioural issues [60,61], which could lead to more dogs being relinquished for rehoming [62].

The main limitation of this study relates to the use of a questionnaire and the use of self-reported data. There is a level of uncertainty in the accuracy of the results. In particular, it is well known that survey respondents tend to over-estimate the amount of activity they perform, which is likely also true for owner estimates of canine activity [5]. Additionally, the study explored owner perceptions of the impact extreme weather had on canine activity, meaning the results are only as reliable as the owner’s perception and recollection of events. The survey method did not permit in-depth exploration of the conflict between owner perceptions of their dog’s ability or willingness to exercise in certain types of weather, and the dog’s actual ability or willingness to exercise in that weather. The results presented here report owner perceptions and are thus likely to be influenced by the owner’s underlying motives relating to their pet’s activity function. Survey participants were recruited via social media and the popular veterinary press, meaning the study likely selected for a demographic of dog owners more actively engaged with their pet’s health. Several large canine sports associations shared the survey, which may have increased the proportion of sport-dog owners in the study. As dog owners are known to be relatively poor at accurately assessing canine body condition score [51], neither body condition score nor an assessment of bodyweight relative to breed was attempted, as the authors felt neither would be reliable measures. As overweight dogs have been found to have increased odds of HRI, future work should aim to address this omission. It was not possible to accurately collect underlying canine health data in the survey, so it is probable that some dogs had conditions that impacted their ability to exercise; however, it is unlikely that the study sample included an over-representation of dogs with underlying health conditions that would impact their ability to exercise, given the relatively high proportion of dogs regularly participating in sports.

The relatively large confidence intervals reported for the statistical models suggest the study is underpowered, and the low numbers of respondents from non-European countries limits the conclusions that can be drawn from analyses including location. Additionally, the reliance upon volunteers offering to translate the survey limited the potential for greater international distribution. Future work could directly measure canine activity levels over a full year to more accurately assess the impact of seasons and extreme weather, and work is urgently needed to assess the safety and efficacy of mitigation actions such as cooling coats to generate better evidence to support owner education.

## 5. Conclusions

This study highlights the impact that extreme weather can have on canine activity levels, suggesting that hot and humid summer weather affects dogs more strongly than cold, wet winter weather. We found that owners of dogs that regularly participated in canine sports were less likely to perceive winter weather as a barrier to canine activity, and were more likely to use mitigation actions such as cooling post exercise and using cooling aids such as cooling coats to facilitate activity in the summer. Reflecting previous studies exploring HRI and owner-reported breed-specific concerns, brachycephalic dogs were considered to be more severely impacted by hot weather. However, they were also considered to be negatively impacted by cold weather, suggesting that brachycephalic dogs may be disproportionately impacted by all forms of extreme seasonal weather. Our findings suggest that owners who are more engaged with their dog’s activity may be better informed on topics such as exercise-induced HRI and appropriate mitigation strategies, highlighting the importance of effective owner-focused educational campaigns to protect canine welfare in the face of climate change.

## Figures and Tables

**Table 1:** Survey questions used to generate a winter canine activity score.

Consider the Following Statements and How They Apply to Your Dog’s Activity in Winter:					
Statement	Strongly Disagree	Somewhat Disagree	Neither Agree nor Disagree	Somewhat Agree	Strongly Agree
I am worried about my dog getting too cold	1	2	3	4	5
I am less willing to take my dog out in poor weather	1	2	3	4	5
My dog can undertake more vigorous activity when it is colder	5	4	3	2	1
My dog is less willing to exercise if it is cold	1	2	3	4	5
My dog is less willing to exercise in the rain	1	2	3	4	5
My dog is less willing to exercise in snow	1	2	3	4	5
My dog is less willing to exercise in icy conditions	1	2	3	4	5
I frequently cut short my dogs exercise as they appear unwilling to go any further	1	2	3	4	5
My dog is more likely to slow down the speed of activity	1	2	3	4	5

**Table 2:** Survey questions used to generate a summer canine activity score.

Consider the Following Statements and How They Apply to Your Dog’s Activity in Summer:					
Statement	Strongly Disagree	Somewhat Disagree	Neither Agree nor Disagree	Somewhat Agree	Strongly Agree
I am worried about my dog getting too hot	1	2	3	4	5
I am less willing to take my dog out in hot weather	1	2	3	4	5
My dog cannot undertake more vigorous activity when it is hot	1	2	3	4	5
I am careful to only exercise when it is cool, e.g., early morning	1	2	3	4	5
My dog is less willing to exercise in the heat	1	2	3	4	5
I frequently cut short my dogs exercise as they appear unwilling to go any further	1	2	3	4	5
My dog is more likely to lie down frequently	1	2	3	4	5
My dog is more likely to pant heavily	1	2	3	4	5
My dog is more likely to slow down the speed of activity	1	2	3	4	5

**Table 3:** The reported daily activity for 1713 adult dogs reported in the survey, compared to the UK Kennel Club recommended daily activity levels by breed. Where the recommendation is more than 120 min per day, the “exceeded” recommendation response is not applicable (N/A).

		Reported Daily Activity Compared to Recommendation (% of Responses)		
Breed	UK Kennel Club Recommendation for Daily Activity (Minutes)	Not Achieved	Achieved	Exceeded
Australian Shepherd Dog	More than 120	76.9	23.1	N/A
Beagle	60	0.0	27.3	72.7
Belgian Malinois Shepherd Dog	More than 120	70.0	30.0	N/A
Border Terrier	60	17.4	17.4	65.2
Boxer	More than 120	81.0	19.0	N/A
Cavalier King Charles Spaniel	60	33.3	33.3	33.3
Chihuahua	30	21.4	50.0	28.6
Cocker Spaniel	60	7.9	30.3	61.8
Collie	More than 120	77.5	22.5	N/A
Dachshund	60	22.2	22.2	55.6
Dalmatian	More than 120	57.1	42.9	N/A
Flat-Coated Retriever	More than 120	84.6	15.4	N/A
French Bulldog	60	21.4	50.0	28.6
German Shepherd Dog	More than 120	79.7	20.3	N/A
German Short-Haired Pointer	More than 120	61.7	38.3	N/A
Golden Retriever	More than 120	94.2	5.8	N/A
Greyhound	60	10.5	36.8	52.6
Jack Russell Terrier	60	21.6	33.3	45.1
Labrador Retriever	More than 120	83.6	16.4	N/A
Papillon	30	0.0	26.1	73.9
Pointer (English)	More than 120	63.6	36.4	N/A
Pug	60	28.6	28.6	42.9
Rottweiler	More than 120	100	0.0	N/A
Shetland Sheepdog	60	18.2	27.3	54.5
Shih-Tzu	60	14.3	35.7	50.0
Siberian Husky	More than 120	77.8	22.2	N/A
Springer Spaniel	More than 120	79.9	20.1	N/A
Staffordshire Bull Terrier	60	20.6	34.8	44.7
Standard Doberman Pinscher	More than 120	81.8	18.2	N/A
Weimaraner	More than 120	69.6	30.4	N/A
West Highland White Terrier	60	10.0	60.0	30.0
Whippet	60	6.7	73.3	20.0
Yorkshire Terrier	30	6.7	46.7	46.7

**Table 4:** Frequency (and percentage) of dogs undertaking different types of activity.

Activity Type	More than Once Daily (%)	Daily (%)	Every Few Days (%)	Weekly (%)	Every Few Weeks (%)	Monthly (%)	Less than Monthly (%)	Never (%)	No Response (%)
High intensity exercise	371 (11.8)	1270 (40.3)	682 (21.6)	254 (8.1)	127 (4.0)	45 (1.4)	137 (4.3)	267 (8.5)	0 (0.0)
Walking on lead	527 (16.7)	976 (31.0)	351 (11.1)	211 (6.7)	143 (4.5)	77 (2.4)	350 (11.1)	423 (13.4)	95 (3.0)
Walking off lead	768 (24.4)	1262 (40.0)	368 (11.7)	163 (5.2)	76 (2.4)	50 (1.6)	111 (3.5)	307 (9.7)	48 (1.5)
Running on lead	43 (1.4)	219 (6.9)	267 (8.5)	175 (5.6)	161 (5.1)	83 (2.6)	333 (10.6)	1693 (53.7)	179 (5.7)
Running off lead	686 (21.8)	1202 (38.1)	383 (12.1)	186 (5.9)	108 (3.4)	59 (1.9)	112 (3.6)	352 (11.2)	65 (2.1)
Playing fetch/retrieving	414 (13.1)	924 (29.3)	609 (19.3)	213 (6.8)	152 (4.8)	71 (2.3)	168 (5.3)	518 (16.4)	84 (2.7)
Swimming outdoors	26 (0.8)	115 (3.6)	315 (10.0)	293 (9.3)	287 (9.1)	167 (5.3)	494 (15.7)	1335 (42.3)	121 (3.8)
Swimming indoors/hydrotherapy	2 (0.1)	0 (0.0)	6 (0.2)	53 (1.7)	40 (1.3)	46 (1.5)	144 (4.6)	2708 (85.9)	154 (4.9)
Other sport participation	23 (0.7)	73 (2.3)	221 (7.0)	338 (10.7)	82 (2.6)	51 (1.6)	85 (2.7)	2010 (63.7)	270 (8.6)

**Table 5:** Variables influencing owner perception of winter weather effects on canine activity score. Factors with a positive B value increased the winter weather effects score and factors with a negative B value decreased the winter weather effects score.

Variable	Sub-Group	N	B	95% Confidence Interval	*p*-Value
Skull shape	Mesocephalic	1811	Reference		
	Brachycephalic	172	0.16	0.11 to 0.21	<0.001
	Brachycephalic cross	57	0.16	0.07 to 0.24	<0.001
	Dolichocephalic	357	0.09	0.05 to 0.12	<0.001
	Not available	504	0.02	−0.01 to 0.05	0.175
Bodyweight (kg)	<10	389	Reference		
	10 to <20	800	−0.14	−0.18 to −0.10	<0.001
	20 to <30	875	−0.18	−0.22 to −0.14	<0.001
	30 to <40	454	−0.20	−0.25 to −0.16	<0.001
	40 to <50	93	−0.23	−0.31 to −0.16	<0.001
	50+	38	−0.32	−0.43 to −0.22	<0.001
	Missing	252	−0.10	−0.15 to −0.05	<0.001
Age (years)	<2	460	Reference		
	2 to <4	669	−0.01	−0.05 to 0.03	0.608
	4 to <6	590	0.00	−0.04 to 0.04	0.930
	6 to <8	440	−0.02	−0.06 to 0.02	0.355
	8 to <10	322	0.05	0.00 to 0.10	0.044
	10 to <12	226	0.06	0.01 to 0.11	0.030
	12 to <14	129	0.11	0.05 to 0.18	0.001
	14+	65	0.12	0.03 to 0.20	0.008
Sex/neuter	Female entire	352	Reference		
	Female neuter	1007	0.04	0.00 to 0.08	0.054
	Male entire	478	0.01	−0.04 to 0.05	0.817
	Male neuter	1064	0.06	0.02 to 0.10	0.004
Sport participation	Yes	792	−0.12	−0.15 to −0.09	<0.001
	No	2109	Reference		
Total daily activity time (minutes)	Up to 10	39	Reference		
	10 to <30	272	−0.03	−0.13 to 0.08	0.653
	30 to <60	899	−0.10	−0.20 to 0.01	0.075
	60 to <120	1185	−0.16	−0.27 to −0.06	0.002
	120 or more	506	−0.19	−0.29 to −0.08	0.001
Continent	Europe	2463	Reference		
	Africa	6	0.19	−0.06 to 0.45	0.139
	Asia	31	−0.04	−0.15 to 0.08	0.531
	Australia	99	0.12	0.06 to 0.19	<0.001
	North America	290	0.08	0.04 to 0.12	<0.001
	South America	12	0.21	0.03 to 0.39	0.025

**Table 6:** Variables influencing owner perception of summer weather effects on canine activity score, variables with a positive B value increased the summer weather effects score and factors with a negative B value decreased the summer effects score.

Variable	Sub-Group	N	B	95% Confidence Interval	*p*-Value
Daily exercise (minutes)	Up to 10	39	Reference		
	10 to <30	272	−0.01	−0.08 to 0.06	0.754
	30 to <60	899	−0.04	−0.10 to 0.03	0.239
	60 to <120	1185	−0.07	−0.13 to 0.00	0.045
	Over 120	506	−0.09	−0.15 to −0.02	0.011
Continent	Europe	2463	Reference		
	Africa	6	−0.07	−0.23 to 0.09	0.378
	Asia	31	0.10	0.03 to 0.17	0.005
	Australia	99	−0.06	−0.10 to −0.02	0.003
	North America	290	−0.02	−0.04 to 0.01	0.141
	South America	12	−0.01	−0.12 to 0.11	0.894
Skull shape	Mesocephalic	1811	Reference		
	Brachycephalic	172	0.08	0.05 to 0.11	<0.001
	Brachycephalic cross	57	0.05	0.00 to 0.10	0.060
	Dolichocephalic	357	−0.02	−0.04 to 0.01	0.130
	Not available	504	0.00	−0.02 to 0.02	0.958
Bodyweight (kg)	<10	389	Reference		
	10 to <20	800	0.01	−0.02 to 0.03	0.548
	20 to <30	875	0.02	0.00 to 0.05	0.062
	30 to <40	454	0.06	0.03 to 0.09	<0.001
	40 to <50	93	0.06	0.02 to 0.10	0.009
	50+	38	0.08	0.01 to 0.15	0.018
	Missing	252	0.00	−0.03 to 0.04	0.850
Sport participation	No	2109	Reference		
	Yes	792	−0.02	−0.04 to 0.00	0.029
Sex/neuter	Female entire	352	Reference		
	Female neuter	1007	0.02	−0.01 to 0.04	0.149
	Male entire	478	0.01	−0.02 to 0.04	0.466
	Male neuter	1064	0.03	0.01 to 0.05	0.013

**Table 7:** Mitigation model 1, use of a coat in winter: canine variables tested through binary logistic regression for their association with coat use in winter to facilitate activity. Increased odds of coat use are indicated by an odds ratio greater than one, and an odds ratio lower than one indicates reduced odds of coat use.

Variable	Sub-Group	N	Odds Ratio	95% Confidence Interval	*p*-Value
Skull	Mesocephalic	1942	Reference		
	Brachycephalic	188	3.23	2.29 to 4.55	<0.001
	Brachycephalic designer hybrid	58	3.66	1.90 to 7.03	<0.001
	Dolichocephalic	385	1.84	1.46 to 2.31	<0.001
	Not applicable	555	1.31	1.08 to 1.59	0.006
Bodyweight (kg)	<10	411	Reference		
	10 to <20	862	0.71	0.55 to 0.92	0.008
	20 to <30	956	0.54	0.42 to 0.70	<0.001
	30 to <40	482	0.33	0.25 to 0.44	<0.001
	40 to <50	107	0.38	0.24 to 0.59	<0.001
	50+	42	0.21	0.11 to 0.42	<0.001
	Missing	268	0.51	0.37 to 0.71	<0.001

**Table 8:** Mitigation model 2, use of cooling after exercise in summer: canine variables tested through binary logistic regression for their association with coat use in winter to facilitate activity. Increased odds of coat use are indicated by an odds ratio greater than one; an odds ratio lower than one indicates reduced odds of coat use.

Variable	Sub-Group	N	Odds Ratio	95% Confidence Interval	*p*-Value
Age (years)	<2	497	Reference		
	2 to <4	717	0.78	0.61 to 1.00	0.047
	4 to <6	632	0.57	0.45 to 0.73	<0.001
	6 to <8	476	0.55	0.43 to 0.72	<0.001
	8 to <10	354	0.60	0.45 to 0.80	<0.001
	10 to <12	244	0.57	0.42 to 0.79	<0.001
	12 to <14	138	0.52	0.35 to 0.78	0.001
	14+	72	0.63	0.38 to 1.06	0.083
Skull shape	Mesocephalic	1944	Reference		
	Brachycephalic	187	1.03	0.75 to 1.40	0.874
	Brachycephalic designer crossbred	59	0.89	0.52 to 1.52	0.674
	Dolichocephalic	385	0.87	0.69 to 1.08	0.209
	Not applicable	555	0.80	0.66 to 0.97	0.025
Daily exercise (minutes)	Over 120	549	Reference		
	60 to <120	1287	0.89	0.72 to 1.10	0.285
	30 to <60	295	0.69	0.51 to 0.93	0.013
	10 to <30	956	0.80	0.64 to 0.99	0.042
	Up to 10	43	0.28	0.14 to 0.57	<0.001
Sport participation	Yes	2288	Reference		
	No	842	1.45	1.23 to 1.72	<0.001

**Table 9:** Mitigation model 3, use of cooling aids (such as cooling coats) in summer: canine variables tested through binary logistic regression for their association with coat use in winter to facilitate activity. Increased odds of coat use are indicated by an odds ratio greater than one; an odds ratio lower than one indicates reduced odds of coat use.

Variable	Sub-Group	N	Odds Ratio	95% Confidence Interval	*p*-Value
Sport participation	No sport participation	2278	Reference		
	Sport participation	842	2.05	1.72 to 2.45	<0.001
Skull	Mesocephalic	1939	Reference		
	Brachycephalic	186	1.98	1.44 to 2.72	<0.001
	Brachycephalic designer crossbred	59	0.95	0.51 to 1.75	0.866
	Dolichocephalic	383	0.84	0.65 to 1.10	0.205
	Not applicable	553	0.86	0.69 to 1.09	0.207

## Data Availability

The data presented in this study are available on request from the corresponding author.

## Appendix A

**Table A1:** Univariable linear regression results for breed type association with winter weather effects on canine activity scores. Sub-groups with a positive B value increased the winter weather effects score and sub-groups with a negative B value decreased the winter effects score.

Variable	Sub-Group	N	B	95% Confidence Interval	*p*-Value
Breed type	Labrador Retriever	471	Reference		
	Yorkshire Terrier	13	0.47	0.29 to 0.65	<0.001
	Whippet	16	0.43	0.27 to 0.59	<0.001
	Chihuahua	14	0.41	0.24 to 0.58	<0.001
	Greyhound	18	0.38	0.23 to 0.54	<0.001
	Pug	21	0.37	0.23 to 0.51	<0.001
	Papillon	23	0.35	0.21 to 0.48	<0.001
	Dachshund	10	0.34	0.13 to 0.54	0.001
	Staffordshire Bull Terrier	128	0.29	0.23 to 0.35	<0.001
	French Bulldog	14	0.29	0.11 to 0.46	0.001
	Cavalier King Charles Spaniel	18	0.29	0.13 to 0.44	<0.001
	Shih-Tzu	13	0.25	0.07 to 0.43	0.006
	Jack Russell Terrier	49	0.24	0.15 to 0.34	<0.001
	Boxer	66	0.17	0.08 to 0.25	<0.001
	West Highland White Terrier	10	0.16	−0.04 to 0.37	0.117
	Beagle	22	0.15	0.01 to 0.29	0.031
	Rottweiler	12	0.14	−0.05 to 0.33	0.137
	Other Designer Hybrid	67	0.14	0.06 to 0.23	<0.001
	Border Terrier	24	0.13	−0.01 to 0.26	0.062
	Lurcher	61	0.12	0.04 to 0.21	0.005
	Crossbreed	539	0.11	0.07 to 0.15	<0.001
	Other Purebred	438	0.10	0.06 to 0.14	<0.001
	Pointer (English)	33	0.03	−0.09 to 0.14	0.625
	Dalmatian	14	0.03	−0.14 to 0.21	0.712
	Weimaraner	51	0.02	−0.07 to 0.12	0.618
	Cocker Spaniel	88	0.01	−0.06 to 0.09	0.768
	Standard Doberman Pinscher	21	0.00	−0.14 to 0.15	0.967
	Golden Retriever	55	0.00	−0.09 to 0.09	0.946
	German Shepherd Dog	78	0.00	−0.08 to 0.08	0.986
	German Short-Haired Pointer	59	−0.01	−0.10 to 0.08	0.792
	Cockapoo	16	−0.01	−0.17 to 0.16	0.929
	Sprocker	10	−0.06	−0.27 to 0.14	0.542
	Collie	144	−0.06	−0.12 to 0.00	0.065
	Springer Spaniel	175	−0.10	−0.16 to −0.04	<0.001
	Shetland Sheepdog	12	−0.10	−0.28 to 0.09	0.313
	Australian Shepherd Dog	11	−0.10	−0.30 to 0.09	0.300
	Working Cocker Spaniel	53	−0.12	−0.21 to −0.02	0.014
	Belgian Malinois Shepherd Dog	10	−0.15	−0.35 to 0.06	0.165
	Flat-Coated Retriever	13	−0.18	−0.36 to 0.00	0.055
	Siberian Husky	11	−0.21	−0.4 to −0.01	0.038

**Table A2:** Univariable linear regression results for breed type association with summer weather effects on canine activity scores. Sub-groups with a positive B value increased the winter weather effects score and sub-groups with a negative B value decreased the winter effects score.

Variable	Sub-Group	N	B	95% Confidence Interval	*p*-Value
Breed type	Labrador Retriever	471	Reference		
	Boxer	66	0.14	0.09 to 0.19	<0.001
	Rottweiler	12	0.11	0.00 to 0.22	0.058
	French Bulldog	14	0.11	0.01 to 0.21	0.035
	Staffordshire Bull Terrier	128	0.09	0.05 to 0.13	<0.001
	Shih-Tzu	13	0.09	−0.02 to 0.19	0.117
	Pug	21	0.07	−0.02 to 0.15	0.127
	Greyhound	18	0.05	−0.05 to 0.14	0.321
	Border Terrier	24	0.05	−0.03 to 0.13	0.201
	West Highland White Terrier	10	0.02	−0.10 to 0.14	0.774
	Weimaraner	51	0.02	−0.03 to 0.08	0.425
	Golden Retriever	55	0.02	−0.03 to 0.08	0.378
	Shetland Sheepdog	12	0.01	−0.10 to 0.12	0.901
	Jack Russell Terrier	49	0.01	−0.05 to 0.07	0.681
	German Shepherd Dog	78	0.01	−0.04 to 0.05	0.772
	Chihuahua	14	0.01	−0.09 to 0.12	0.799
	Yorkshire Terrier	13	0.00	−0.11 to 0.11	0.994
	Papillon	23	0.00	−0.08 to 0.08	0.973
	Other Designer	67	0.00	−0.05 to 0.05	0.897
	Lurcher	61	0.00	−0.05 to 0.05	0.926
	Standard Doberman Pinscher	21	−0.01	−0.10 to 0.07	0.764
	Crossbreed	539	−0.01	−0.03 to 0.02	0.488
	Australian Shepherd Dog	11	−0.01	−0.13 to 0.11	0.866
	Other Purebred	438	−0.02	−0.04 to 0.01	0.252
	Flat-Coated Retriever	13	−0.02	−0.13 to 0.08	0.680
	Sprocker	10	−0.03	−0.15 to 0.10	0.687
	Siberian Husky	11	−0.03	−0.15 to 0.09	0.599
	German Short-Haired Pointer	59	−0.03	−0.08 to 0.02	0.243
	Working Cocker Spaniel	53	−0.04	−0.10 to 0.01	0.145
	Cocker Spaniel	88	−0.05	−0.09 to 0.00	0.047
	Cockapoo	16	−0.05	−0.15 to 0.04	0.289
	Beagle	22	−0.06	−0.14 to 0.02	0.150
	Whippet	16	−0.07	−0.17 to 0.03	0.149
	Springer Spaniel	175	−0.07	−0.11 to −0.04	<0.001
	Collie	144	−0.07	−0.11 to −0.04	<0.001
	Dalmatian	14	−0.08	−0.19 to 0.02	0.114
	Cavalier King Charles Spaniel	18	−0.08	−0.17 to 0.01	0.084
	Dachshund	10	−0.09	−0.21 to 0.03	0.141
	Belgian Malinois Shepherd Dog	10	−0.12	−0.24 to 0.01	0.060
	Pointer (English)	33	−0.14	−0.20 to −0.07	<0.001

## Appendix B

**Table A3:** Univariable logistic regression results for breed type associated with coat use in winter. Increased odds of coat use are indicated by an odds ratio greater than one, an odds ratio lower than one indicates reduced odds of coat use.

Variable	Sub-Group	Odds Ratio	95% Confidence Interval	*p*-Value
Breed type	Labrador Retriever	Reference		
	Whippet	26.69	3.51 to 202.88	0.002
	Greyhound	14.18	3.24 to 62.05	<0.001
	Cavalier King Charles Spaniel	13.35	3.04 to 58.68	0.001
	Yorkshire Terrier	10.85	2.42 to 48.57	0.002
	Shih-Tzu	10.01	2.22 to 45.20	0.003
	Lurcher	8.84	4.40 to 17.79	<0.001
	Chihuahua	6.67	1.86 to 23.95	0.004
	Papillon	6.34	2.33 to 17.25	<0.001
	Other Designer Hybrid	5.30	2.99 to 9.40	<0.001
	Pointer (English)	4.82	2.21 to 10.50	<0.001
	French Bulldog	4.59	1.44 to 14.61	0.010
	West Highland White Terrier	3.89	1.00 to 15.23	0.051
	Standard Doberman Pinscher	3.81	1.54 to 9.44	0.004
	Pug	3.34	1.40 to 7.94	0.006
	Boxer	3.34	2.00 to 5.57	<0.001
	Jack Russell Terrier	3.06	1.68 to 5.58	<0.001
	Weimaraner	2.67	1.49 to 4.80	0.001
	German Shepherd Dog	2.60	1.53 to 4.44	<0.001
	Dachshund	2.50	0.70 to 8.98	0.159
	Staffordshire Bull Terrier	2.47	1.70 to 3.61	<0.001
	Dalmatian	2.23	0.76 to 6.51	0.144
	Cocker Spaniel	2.17	1.38 to 3.40	0.001
	Crossbreed	1.95	1.53 to 2.48	<0.001
	Working Cocker Spaniel	1.73	1.00 to 2.99	0.051
	Other Purebred	1.58	1.23 to 2.04	<0.001
	Beagle	1.53	0.66 to 3.53	0.320
	Sprocker	1.43	0.47 to 4.32	0.526
	Cockapoo	1.34	0.52 to 3.44	0.550
	Collie	1.05	0.72 to 1.52	0.808
	Shetland Sheepdog	0.83	0.25 to 2.81	0.770
	Border Terrier	0.83	0.35 to 1.99	0.682
	Springer Spaniel	0.79	0.55 to 1.13	0.191
	Rottweiler	0.67	0.21 to 2.16	0.499
	German Short-Haired Pointer	0.60	0.36 to 1.01	0.055
	Australian Shepherd Dog	0.50	0.14 to 1.84	0.298
	Golden Retriever	0.48	0.25 to 0.92	0.026
	Siberian Husky	0.33	0.07 to 1.54	0.159
	Flat-Coated Retriever	0.28	0.06 to 1.26	0.096
	Belgian Malinois Shepherd Dog	0.19	0.02 to 1.47	0.111

**Table A4:** Univariable logistic regression results for breed type associated with post exercise cooling in summer. Increased odds of coat use are indicated by an odds ratio greater than one, an odds ratio lower than one indicates reduced odds of coat use.

Variable	Sub-Group	Odds Ratio	95% Confidence Interval	*p*-Value
Breed type	Labrador Retriever	Reference		
	French Bulldog	7.92	1.03 to 60.72	0.046
	Springer Spaniel	1.75	1.19 to 2.57	0.004
	Working Cocker Spaniel	1.45	0.79 to 2.66	0.229
	Rottweiler	1.41	0.44 to 4.57	0.563
	Boxer	1.34	0.79 to 2.27	0.283
	West Highland White Terrier	1.32	0.34 to 5.17	0.690
	Australian Shepherd Dog	1.27	0.39 to 4.19	0.691
	Cocker Spaniel	1.25	0.78 to 2.01	0.359
	German Short-Haired Pointer	1.13	0.69 to 1.84	0.620
	Shetland Sheepdog	1.13	0.34 to 3.81	0.842
	Dalmatian	1.02	0.34 to 3.08	0.974
	Golden Retriever	1.00	0.57 to 1.75	0.991
	Lurcher	0.98	0.57 to 1.70	0.953
	Sprocker	0.91	0.29 to 2.81	0.863
	German Shepherd Dog	0.88	0.52 to 1.51	0.646
	Other Designer Hybrid	0.81	0.49 to 1.34	0.411
	Collie	0.80	0.55 to 1.15	0.223
	Border Terrier	0.79	0.35 to 1.82	0.583
	Staffordshire Bull Terrier	0.79	0.54 to 1.16	0.226
	Cockapoo	0.78	0.31 to 1.97	0.596
	Flat-Coated Retriever	0.75	0.26 to 2.21	0.607
	Standard Doberman Pinscher	0.68	0.29 to 1.60	0.377
	Pug	0.67	0.29 to 1.52	0.338
	Crossbreed	0.64	0.51 to 0.82	<0.001
	Greyhound	0.63	0.25 to 1.58	0.322
	Weimaraner	0.61	0.34 to 1.08	0.092
	Other Purebred	0.58	0.45 to 0.75	<0.001
	Belgian Malinois Shepherd Dog	0.57	0.16 to 1.98	0.373
	Dachshund	0.57	0.16 to 1.98	0.373
	Papillon	0.57	0.25 to 1.29	0.173
	Siberian Husky	0.57	0.18 to 1.78	0.330
	Jack Russell Terrier	0.54	0.31 to 0.97	0.039
	Pointer (English)	0.53	0.27 to 1.06	0.074
	Chihuahua	0.50	0.18 to 1.39	0.181
	Beagle	0.47	0.20 to 1.11	0.086
	Whippet	0.40	0.15 to 1.06	0.065
	Yorkshire Terrier	0.31	0.10 to 0.95	0.041
	Cavalier King Charles Spaniel	0.28	0.10 to 0.77	0.013
	Shih-Tzu	0.23	0.07 to 0.73	0.013
